# The metabolic syndrom and the prescription of psychotropic drugs in children and adolescents: three clinical cases

**DOI:** 10.1192/j.eurpsy.2023.1577

**Published:** 2023-07-19

**Authors:** Z. Elmaataoui, H. Belhadga, H. Kisra

**Affiliations:** Hospital Ar-Razi of Sale, Sale, Morocco

## Abstract

**Introduction:**

Antipsychotics have shown their interest in several pathologies of the child and the adolescent. However, in this vulnerable population, they are not without adverse effects. Depending on the type of molecule used, classical neuroleptics or second generation antipsychotics, but also within these own classes, the profile of tolerance and adverse effects differs. In this sense, children treated with psychotropic drugs have a higher risk of developing metabolic syndrome compared to children who do not take this treatment.

**Objectives:**

The aim of this work is to discuss the metabolic syndrome in children treated with psychotropic drugs and this through three clinical vignettes.

**Methods:**

we conducted our study through an analysis of three clinical cases

**Results:**

It is about three children followed in the service of child psychiatry of the hospital Ar-razi of salé, aged respectively 11, 13 and 14 years, these children were put under psychotropic drugs for various mental disorders and developed during the evolution of metabolic side effects in particular a dyslipidemia, a diabetes of type 2 revealed by a diabetic ketoacidosis and a hyperprolactinemia.

**Conclusions:**

Systematic monitoring and preventive programs targeting weight gain and metabolic side effects should be an integral part of the overall management of adolescents on psychotropic medications.

**Disclosure of Interest:**

None Declared

